# Hepatocellular carcinoma in Hepatitis B and Human Immunodeficiency Virus coinfection in Africa: a focus on surveillance

**DOI:** 10.20517/2394-5079.2022.32

**Published:** 2022-10-14

**Authors:** Qian Wan, Chimaobi Anugwom, Hailemichael Desalegn, Jose D. Debes

**Affiliations:** 1 Department of Medicine, Division of Infectious Disease and International Medicine & Division of Gastroenterology, University of Minnesota, Minneapolis, MN 55455, USA.; 2 Health Partners Digestive Care, Minneapolis, MN 55455, USA.; 3 Saint Paul Millennium Hospital, Addis Ababa 1000, Ethiopia.; 4 Arusha Lutheran Medical Centre, Arusha, Tanzania.

**Keywords:** HBV, HIV, coinfection, hepatocellular carcinoma, surveillance, Sub-Saharan Africa

## Abstract

Human immunodeficiency virus (HIV) and hepatitis-B virus (HBV) infections are weighty public health challenges, especially in the African continent. The direct carcinogenic effect of HBV means that it remains a potent cause of early-onset hepatocellular carcinoma (HCC) in Sub-Saharan Africa (SSA), where it causes significant morbidity and mortality. The presence of HIV infection in HBV-infected patients poses a complicating factor, as coinfection has been shown to hasten the progression of liver disease to cirrhosis and HCC, and often resulting in early-age hepatocarcinogenesis with consequent late diagnosis and lower survival. In this review, we discuss this unique conundrum, the epidemiology of HIV-HBV coinfection in SSA, its effect on liver disease and development of HCC, as well as practices and barriers to HCC surveillance in this distinct population. We propose a way forward to curb this considerable health burden focusing on reduction of disease stigma, the need for easy-to-measure biomarkers, and implementation of large prospective studies in this population.

## INTRODUCTION

Hepatocellular carcinoma (HCC) is the third cause of cancer-related mortality worldwide, with a large proportion of these deaths occurring in Africa^[1]^. HCC typically develops in the context of liver cirrhosis secondary to underlying chronic liver disease. Chronic infection with Hepatitis B virus (HBV) is the leading cause of HCC, causing more than half of the world’s HCC deaths^[2]^. Infection with the human immunodeficiency virus (HIV) is also a major public health hazard across the world^[3]^. HBV and HIV coinfection is relatively common in endemic areas due to their similar transmission patterns, and previous research has demonstrated that these diseases can lead to worse overall outcomes when co-existing in a patient^[4]^. Moreover, beyond coinfection with HBV, HIV-positive individuals are at a higher risk of developing HCC compared to the HIV un-infected population and can do so at earlier ages^[5]^. Africa and East Asia are estimated to account for more than 75% of global HCC patients^[6]^. Indeed, unique risk factors, such as a high prevalence of HIV in the context of HBV or hepatitis C (HCV) coinfection, as well as exposure to aflatoxins and underreported consumption of alcohol help explain the high incidence of HCC in these regions^[6–8]^. Moreover, the dynamics related to poor outcomes in HCC are increased in resource-limited settings including many parts of Africa, where most of HIV infections occur^[9,10]^. Surveillance for HCC, aimed at early detection and implementation of tumor cure, brings novel complexities when related to HIV-infected individuals. Indeed, recent studies show that ultrasound surveillance has a low performance for HCC in HIV-infected individuals, with a suboptimal 43% rate for early-stage diagnosis, compared to ~63% found in studies in non-HIV-infected cohorts^[11,12]^. Thus, the burden of HCC in HIV and HBV-coinfection as well as the lack of effectiveness of ultrasound for early detection of HCC in these patients all intersect in complexity further augmenting morbidity and mortality in this population. In this review, we aim to address the need and critical role of surveillance of HCC in HIV and HBV coinfected populations, with a focus on the African region.

## HIV-HBV COINFECTION

### Overall epidemiology

**Figure 1.**
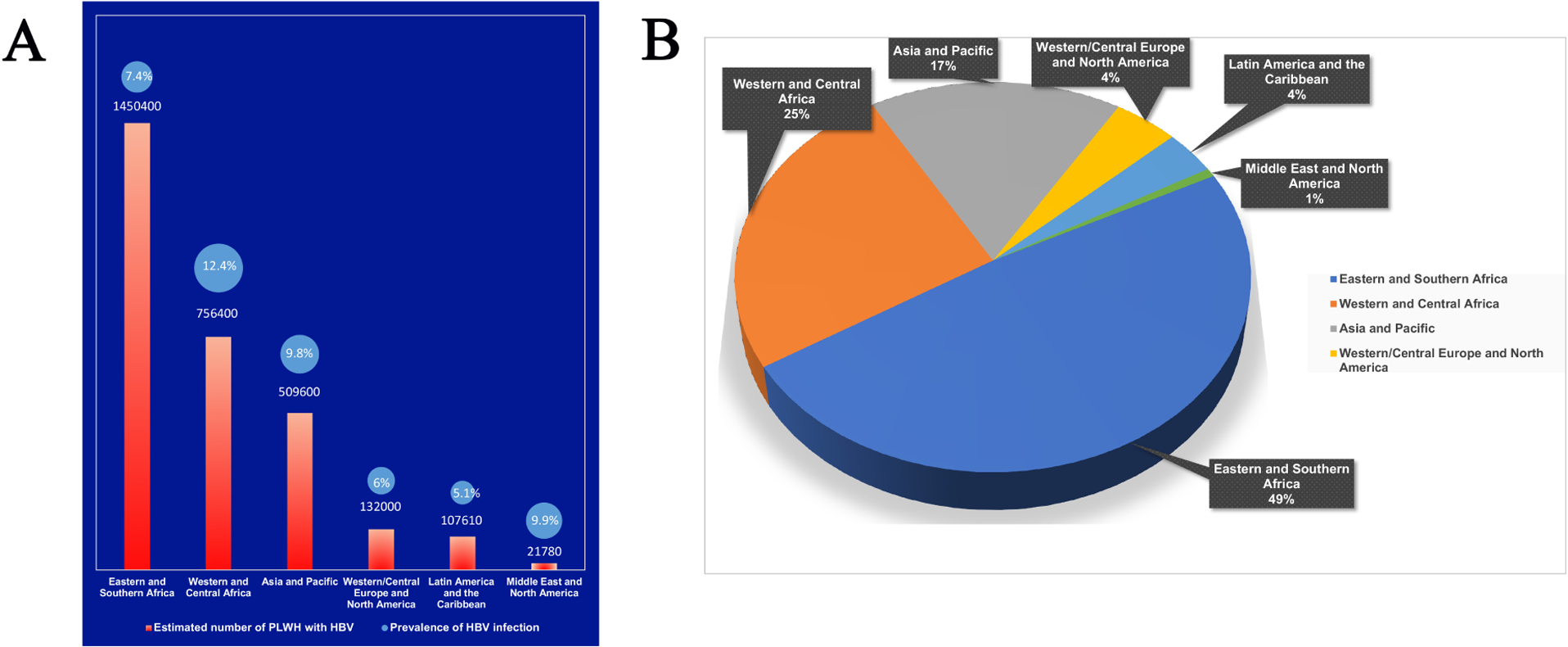
Regional estimates of Hepatitis B Virus infection in People Living with Human Immunodeficiency Virus (PLWH). All the data in the charts were obtained from Leumi *et al*.’s publication^[14]^ in 2020. Note: Eastern Europe and Central Asia’s data is not available. (A). Estimated number of PLWH with HBV infection in different regions of the world and the prevalence of HBV infection in PLWH. (B). Proportion among total regionally estimated HBV infection cases in PLWH. Note: Sub-Saharan Africa (Western, Central, Eastern, and Southern Africa) = 74%

According to a report in 2005, an estimated 2–4 million people infected with HIV are also chronically infected with HBV^[13]^. A 2020 meta-analysis showed an estimate 8.4% coinfection rate of HBV in People Living with HIV (PLWH), with a quarter of those patients having active transmission of the virus^[14]^. The proportion and risk factors of HBV-HIV coinfection differ considerably based on geographical location [Figure 1A]. Due to common transmission channels and associated risk factors, chronic HBV infection in PLWH is more common in areas where both viruses are prevalent, with 74% of estimated regional HBV cases in PLWH coming from Sub-Saharan Africa (SSA) and 17% from the Asia-Pacific region^[14]^ [Figure 1B]. Because of perinatal and early childhood transmission patterns, SSA accounts for the majority (65%) of HIV infections worldwide and has a higher incidence of chronic HBV infection^[14]^.

### HIV-HBV coinfection epidemiology in Africa

The World Health Organization (WHO) African Region is the region most affected by HIV in the world, with 25 million people living with the virus. This region does not only rank the highest in prevalence, but it also has the highest number of new infections, at 1.1 million people in 2018. The countries with the highest rates of HIV in Africa include Eswatini, Lesotho, and Botswana. In 2020, Eswatini had the highest prevalence of HIV with a rate of almost 27%^[15]^. Looking at coinfection, from a total of 37 million people who were infected with HIV, 2.7 and 2.3 million people are estimated to be coinfected with HBV and HCV, respectively^[16]^.

Among countries in SSA, there is significant variability in the prevalence of HBV/HIV coinfection. A study from Ethiopia determined the HBV/HIV coinfection rate at 5.5%, somewhat similar to that in Uganda at 6.7% and in Tanzania’s at 6.6%. In contrast, some reports expose higher coinfection rates in other countries such as Sudan (14%) and Cameroon (25%)^[17–21]^. A systematic review by Stabinki *et al*., including 81 studies worldwide, revealed that HIV/HBV coinfection rates differ greatly across SSA^[22]^, varying from 0% to 28% with a median of 7.8%. However, most of the studies described above are not from standardized population-based surveillance, so the estimates may not reflect the full population prevalence. A recently published meta-analysis including 314 studies (1999–2022) by Kenfack-Momo *et al*. calculated the HBV seroprevalence at 10.5% in PLWH in Africa^[23]^. A subgroup analysis showed that the top three countries in terms of HBV prevalence in HIV were Burkina Faso (14.7%), Nigeria (13.3%) and Ivory Coast (13.1%). It is important to note that substantial heterogeneity exists since the prevalence were estimates calculated from multiple studies^[23]^.

### The effect of HIV-HBV coinfection on the liver

HIV can affect HBV and liver Physiology in various ways. The presence of HIV infection may increase transmission and reduce control of HBV. In mothers infected with HBV, transmission from mother to child is aided by the presence of HIV^[24]^. Moreover, HIV is a risk factor for reactivation of HBV, particularly in patients with severe immunosuppression^[25]^. There is also evidence showing that interaction between HIV and HBV reduces the ability to eliminate HBV infection after exposure, resulting in increased HBV DNA concentrations, which in turn, leads to rapid progression to fibrosis and HCC^[26]^.

There is also a direct effect of HIV on HBV and liver physiology. Recent research has linked rapid progression to cirrhosis from a direct impact of HIV on stellate cells, which play an essential role in the development of liver fibrosis^[27,28]^. The poor clearance of HBV due to the presence of HIV may potentiate the deleterious effect of unchecked HBV replication. It has been shown that HIV-HBV coinfection results in higher morbidity and mortality than HIV infection alone, thus lending credence to a synergistic effect^[29]^. Also, when compared to their HIV-negative counterparts, people with HIV-associated HCC present at younger ages, implying a shorter latency to hepatocarcinogenesis. Furthermore, those who are HIV and HBV coinfected, particularly those with low CD4+ cell numbers, are at an elevated risk of liver-related death^[30,31]^.

Finally, treatment of HIV may affect HBV and liver physiology. Antiretroviral therapy (ART) has improved HIV patient survival by extending life expectancy. An expected consequence of this, is the dramatically increased risk of developing complications from other associated chronic diseases, such as cirrhosis and HCC^[32]^. Patients on ART, on the other hand, have a higher risk of hepatotoxicity after starting the therapy^[33]^. Novel ART has decreased the toxic effect of these medications in the liver. Indeed, the use of new therapeutic agents in combination with tenofovir (the most commonly used antiviral against HBV in PLWH) have less deleterious effect on steatosis and they are thought to potentially decrease risk of HCC by decreasing the risk of cirrhosis. Nonetheless, the specific effect of ART on HCC has not been well studied and many countries in Africa still use relatively older treatments which can increase the risk of liver dysfunction^[23,34]^.

## HEPATOCELLULAR CARCINOMA

### HCC in people with HIV infection

PLWH have an increased risk of malignancies, often termed HIV-associated cancers. A registry linkage study of over four hundred thousand PLWH documented 21,294 incident cancers with a standardized incidence ratio of 1.69^[35]^. Moreover, three specific malignancies - Kaposi sarcoma, invasive cervical cancer and aggressive B-cell non-Hodgkin lymphoma are AIDS-defining cancers^[36]^.

The increased cancer incidence in people with HIV is related to multiple considerations. Firstly, HIV infection results in a loss of immune function and poor regulation of B-cells, leading to poor response when infections with oncogenic viruses occurs. This, in addition to the chronic B-cell activation seen even in controlled HIV infection may be responsible for development of lymphomas^[37,38]^. Secondly, a few oncogenic viruses have a similar mode of transmission to HIV. Such oncoviruses include HBV and HCV. Finally, as mentioned above, the improved care of patients with HIV has resulted in patients living longer with typical malignancies such as colorectal, breast and prostate cancer being seen more frequently in these group of patients^[39]^.

HCC remains an important cause of morbidity and mortality worldwide and it is associated with multiple diverse etiologic factors, especially HBV and HCV^[40]^. In PLWH, HCC is thought to occur at increased rates even without any underlying liver disease^[41]^. A large study using the national Veterans Health Administration Database, compared subjects with HIV alone with those with HIV-HCV coinfection and found that the presence of HCV dramatically increased the risk of development of cirrhosis and HCC^[42]^. The effect of HCV on the development of HCC in patients with HIV was also shown in a study of three cancer registry databases in Italy^[5]^. In SSA, it is thought that the prevalence of HBV infection in PLWH who develop HCC is even more pronounced^[10,40]^. Furthermore, a case-controlled study conducted in referral hospitals of four countries across West Africa revealed an adjusted OR of 2.2 [95%CI: 1.0–5.8] for HCC in HIV indicating a significant correlation between HIV infection and primary liver cancer^[43]^.

**Figure 2.**
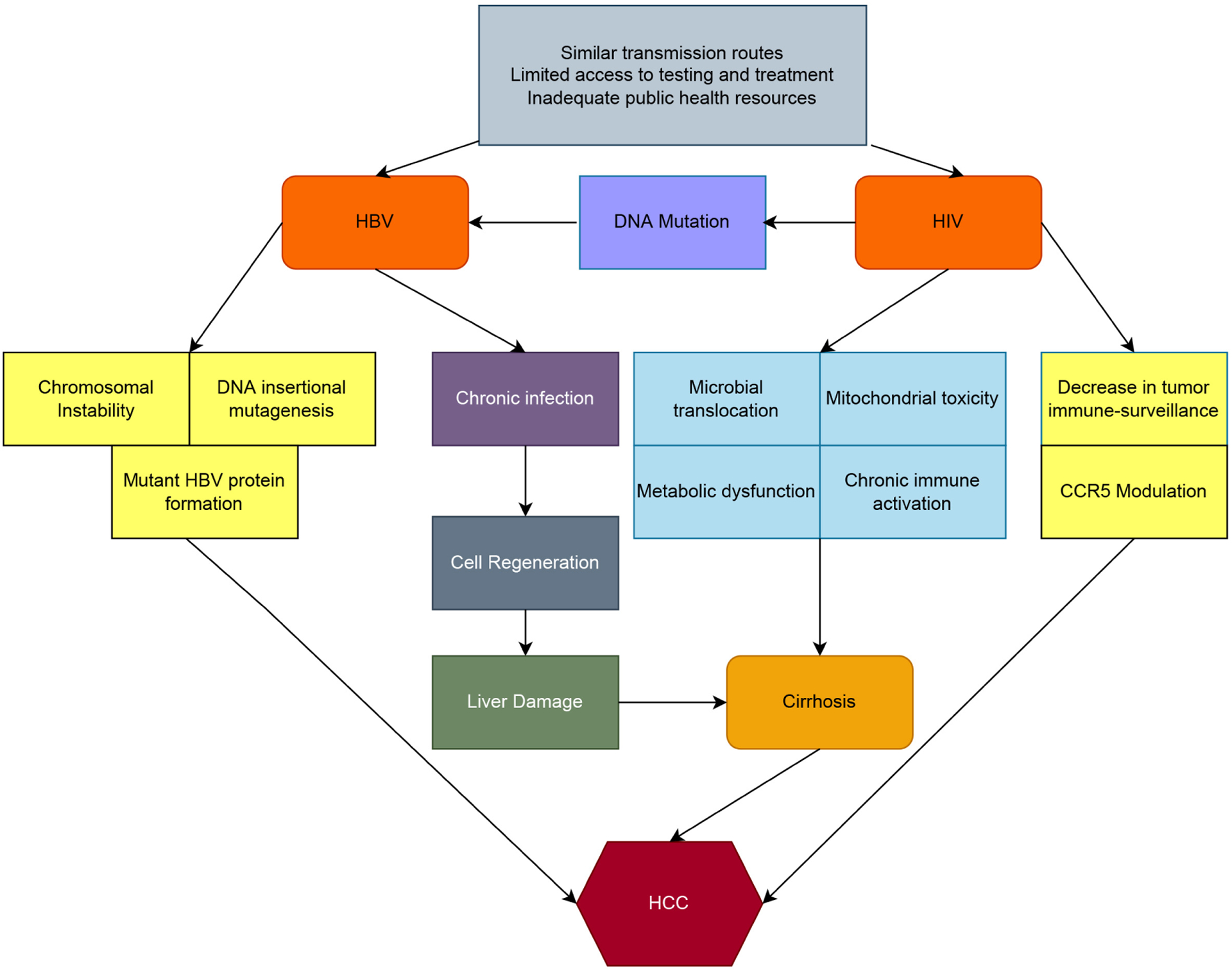
Mechanisms and factors behind HBV-HIV coinfection in the development of cirrhosis and hepatocellular carcinoma in Africa. Flow chart detailing how similar routes of transmission and limited health resources increase the prevalence of HIV-HBV coinfection in Africa. HBV and HIV both induces cirrhosis by damaging liver cells by multiple mechanism, including chronic inflammation in the case of HBV and multiple metabolic alterations in the case of HIV. In addition, HBV by itself can also induce HCC in absence of cirrhosis by direct DNA mutagenesis thought to be related to viral insertion and HIV can increase the risk of HCC by itself by decreasing immunesurveillance (affecting NK cells) and by modulation CCR5, which has been involved in HCC formation.

Multiple factors affect the pathogenesis of HCC in PLWH, especially in those with HBV [Figure 2]. The risk of coinfection with HBV is increased given the similar modes of transmission as alluded to earlier. More interestingly, there seems to be a difference in the pathophysiology of HCC in these patients. Defective CD4 and CD8 T-lymphocyte activity and immune dysregulation leads to unchecked replication of HBV in turn leading to early chronic inflammation, liver fibrosis and consequently, HCC^[4]^. This effect of HIV in the immune system has been buttressed by studies showing increased risk of HCC development in patients exposed to prolonged viremia and to extended periods of lower CD4 T-lymphocyte counts^[44]^.

Interestingly, differential modulation of CCR5 in HIV patients may allow the virus not only to enter CD4 + cells but also to attach to hepatocytes and cause pro-inflammatory and pro-fibrotic cytokine responses. A different study found that CCR5 inhibition dramatically lowers tumor load and fibrosis in a mouse model of HCC^[45]^. This data suggests that HIV may directly affect liver cells and induce HCC through modulation of CCR5^[45]^. Furthermore, reports suggest a more aggressive HCC phenotype in those with HIV as various studies have showed occurrence of malignancy in younger patients and often, at a more advanced stage of disease associated with invasive, infiltrating neoplasms and extrahepatic nodal metastasis^[46,47]^. Although the advanced disease and lower rates of survival may be related to a delayed diagnosis of HCC in these patients, a putative mechanism for aggressive tumor characteristics is likely the presence of HIV-induced growth signals that enhances HCC cell proliferation coupled with the inability of a weakened immune system to mount a potent anti-tumor response^[5]^. These phenomena call for a more proactive stance in the prevention and management of HCC in these patients. Interestingly, the majority of antiretroviral medications used to treat HIV have the inherent potential to be hepatotoxic. A study found out that cumulative use of stavudine (d4T), didanosine (ddI), and amprenavir (APV) was independently linked to higher risk of HCC^[48]^. However, other studies demonstrated that antiretroviral therapy’s benefit on alleviating liver cirrhosis outweighing their role in hepatotoxicity^[49,50]^. Finally, multiple studies have shown that genetic mutations related to increased risk of HCC are more common on HIV-HBV coinfected patients^[51,52]^. Further details on mechanisms of hepatocarcinogenesis in this population can be found in other reviews^[53,54]^. Therapy for HCC in co-infected patients is similar to that of HIV-negative individuals. Moreover, liver transplantation which was in the past not available for this group of patients is now approved in many western countries^[55]^. However, transplantation is still beyond the reach of the great majority of the African continent.

### HCC in HIV-HBV coinfected patients in Africa

A few studies from Africa attempting to explore the connections between HCC and HIV-HBV coinfection have been documented. However, they are limited in number and depth. A study from Mozambique found that 18% of HCC patients (34 cases) enrolled were infected with HIV, and the majority of these patients (68%) were coinfected with HBV. In addition, HCC presented early in HIV-HBV coinfected patients compared to HIV negative individuals^[56]^. A study from South Africa demonstrated that females with HIV-HBV coinfection present at a younger age (36 years) compared to HBV mono-infected women (50 years); more importantly, the HIV-infected patients had a lower median survival (82 days) compared to patients without HIV (181 days)^[10]^. Similarly, a prospective study carried out in a Hospital in Nigeria, showed that most patients with HIV and HCC were HBsAg positive, and at three months, the chances of survival in HIV-infected and HIV-uninfected individuals were 22% and 47%, respectively^[57]^. A recent report from Ethiopia by Tassachew *et al*. studied a large cohort of patients with liver disease and concluded that HCC patients had higher rates of HIV and HIV/HBV co-infection when compared to patients without HCC (8.6% *vs*. 1.8%) and (3.9% *vs*. 0.9%), respectively^[58]^. Interestingly, a separate study from West Africa suggested that HIV infection is not necessarily associated with HCC development^[59]^. All these studies need validation as they are quite limited in their sample size and have been performed in single geographic regions.

## HCC SURVEILLANCE IN HIV-HBV COINFECTED PATIENTS

### Current HCC surveillance

An effective cancer surveillance program aims to reduce cancer-related mortality. As it pertains to HCC, the main goal of surveillance is to detect HCC at stages where successful curative therapies can be applied. When compared to the general population, HIV-infected individuals have a five- to six-fold increased risk of developing HCC^[60]^. Therefore, it is crucial to establish an effective surveillance system in the HIV-infected population.

Surveillance for HCC is currently performed with ultrasound (US) imaging, with or without alpha-fetoprotein (AFP) measurement in blood; and it is recommended by liver and gastroenterology professional societies that this is done every 6 months^[61]^. All guidelines recommend initiating HCC screening for all African-origin HBV-infected patients at earlier ages, regardless of the presence of cirrhosis^[61–63]^. However, despite a thorough examination of the optimal screening frequency, there is a lack of compliance and adherence to this HCC surveillance guidelines in the overall population, as well as in the HIV infected population^[40]^. A variety of factors are likely to contribute to patients’ poor adherence to HCC surveillance in HIV. Healthcare providers may lack knowledge of practice guidelines, and there may be a lack of a standardized system for scheduling and following screening examinations geared toward coinfection care^[64]^.

### HCC surveillance studies in HIV-HBV coinfected populations worldwide

According to a cohort study carried on Paris in 2012, 61% of HCCs in individuals coinfected with HIV and HBV were diagnosed during surveillance programs, compared to 85% of cases without HIV. Despite the difference not being statistically significant (*P* = 0.09) this study does suggest a lower number of HCC diagnosed via surveillance in HIV-HBV coinfected individuals than those with HIV alone^[65]^. Another study in the UK looked at HCC surveillance in HIV-HBV coinfected individuals of African Ancestry and found that only 24% underwent annual assessments and 7% underwent six-monthly assessments as indicated by the guidelines^[66]^. Similarly, a large European cohort of HIV/HBV/HCV coinfected individuals with cirrhosis followed over a period of 10 years found a strikingly low adherence of between 5% and 18% to HCC surveillance guidelines^[67]^. Even though compliance with HCC surveillance standards increased marginally when the duration between US monitoring was prolonged to 12-month in the study, compliance rates remained low, at a maximum of 30%^[67]^. In another study from Canada, only 36% of HIV/HBV coinfected patients seen in HIV practices completed HCC surveillance over a 2-year period, compared to 81% of HBV mono-infected patients seen in hepatology practices^[68]^. Sadly, poor compliance with surveillance adherence was linked to impaired liver function, more advanced tumor stages, larger nodules, and a lower chance of survival^[65]^. This study, however, should be interpreted with caution as the surveillance rate in HBV mono-infected was quite higher than most studies worldwide^[68]^.

Berretta *et al*. in a retrospective review study compared 104 HIV-infected patients and 484 uninfected patients with HCC^[47]^. The results showed that HCC was diagnosed more frequently through screening programs in HIV-uninfected patients than in HIV-infected patients. As expected, diagnosed while in a surveillance program was an independent factor associated with HIV patients’ survival^[67]^. These and other studies, clearly show a decrease in surveillance adherence in HIV-infected patients when compared to HIV-negative ones. This is concerning when adherence rates for HCC surveillance are already low in HIV-negative cohorts.

### HCC surveillance in the HBV-HIV population in Africa

Patients with HCC in Africa have among the lowest reported survival rates in the world, particularly when compared to patients from higher income countries^[69,70]^. A major contributor to this wide disparity in mortality is late presentation and diagnosis, in addition to a relative paucity of disease screening and HCC surveillance. There are no continental bodies or specific guidelines in Africa regarding HCC surveillance in patients with HBV with or without HIV, and approach to surveillance is usually performed by adapting guidelines from other continents. This leads to a lack of uniform approach that serves specific needs of the continent and therefore decreases the uptake on such guidelines. This in turn leads to less screening for HCC in at risk populations (as these populations are not clearly defined in Africa) and therefore late diagnosis. This issue is further aggravated in HIV-coinfected populations. In addition, HCC in African patients can occur at a much earlier age, especially in HBV which makes up the etiology of underlying liver disease in up to 55% of cases^[69]^. Chronic exposure to aflatoxins - a common contaminant of grain products in SSA; and African iron overload syndrome - a consequence of local alcoholic beverage preparation; also contribute to the burden of HCC in this region and may further complicate the clinical course of those with HBV/HIV^[71,72]^. Late diagnosis, in turn leads to limited treatment options, as surgical resection, liver transplantation and curative ablative therapies are largely beneficial only in early stages of HCC^[61]^. Moreover, providers caring for PLWH tend to focus on infection-related issues, and frequently can “omit” the higher risk of HCC that this population has. These problems have been demonstrated in multiple studies across the region. A Ugandan study found that late treatment is responsible for high one-month mortality rate (73%), as monitoring for HCC in patients with cirrhosis is not routinely performed^[73]^. In South Africa, many HBV infections aren’t discovered in PLWH until these individuals are diagnosed with HCC. As a result, many individuals appear with several lesions or large liver lesions on radiological examination, thus limiting the treatment options to palliative strategies^[8]^. Similarly, according to one study carried out in Egypt there is no efficient screening strategy for HCC in cirrhotic individuals with HIV, with only ~10% of new HCC detected during planned screening, while 38% of these patients had multiple liver tumors, and 72% had tumors greater than 5 cm in diameter^[74]^.

It is therefore conceivable that implementing strong surveillance programs in SSA will curb the poor outcomes associated with HCC in those with HBV/HIV. Recently, Riebensahm *et al*. performed a prospective pilot study where 303 HIV-HBV coinfected adults undergoing ART were screened for HCC in two clinics in Zambia^[75]^. In this study, review of the ultrasounds showed that 24% of individuals had periportal fibrosis, 2% cirrhosis and 1% had liver steatosis. Liver lesions were found in 1.8 % of the individuals, but none of these individuals with liver lesions had an elevated serum AFP and not all liver lesions were HCC^[75]^. However, this study suggests that with proper organization, HCC surveillance is possible in this setting.

A myriad of problems affects HCC surveillance in patients with HBV/HIV. Two particular problems in SSA are that of cost and resource availability. Ultrasound is commonly accessible and less expensive, compared to other high-accuracy diagnostic imaging techniques such as computed tomography and magnetic resonance imaging. Nonetheless, adherence to surveillance is a concern due to affordability of multiple visits by patients. Another factor is the operator-dependent nature of ultrasound. The sensitivity of ultrasound in detecting early, small liver lesions is about 45% and so it is recommended that surveillance be carried out in specialized centers by well trained technicians and clinicians^[76]^. Although the potential provision of portable ultrasound machines offers an opportunity to mitigate problems associated with availability, we may still be besieged by issues surrounding technical manpower as training of personnel across the region requires a concerted and comprehensive effort^[77]^. In addition, most studies looking at ultrasound for HCC surveillance do not include HIV-HBV coinfected patients, further undermining the potential of this approach in detecting early HCC. Patient hesitancy and distrust is also a considerable factor - an author of this paper once had a patient (in Africa) tell him that he did not like to go to the best imaging centers in town for surveillance because “they really try hard to find something in the liver”. These factors, either alone or in combination, make the implementation of a surveillance program in SSA very complex.

## FUTURE DIRECTIONS

Despite current recommendations, the implementation of ultrasound as surveillance strategy in HIV-HBV coinfected individuals in Africa clearly falls far short of what is required. Moreover, despite AFP being the strongest studied biomarker, it is by itself insufficient for surveillance, particularly in the setting of HCC related to viral hepatitis, the most common underlying etiology in Africa. All these complexities are amplified by coinfection with HIV as very few studies have addressed either screening tool in this setting. The scientific and public health communities need novel, easy-to-measure biomarkers that could be easily implemented and used across different settings in these populations. A few novel biomarkers, such as desgamma carboxy prothrombin (DCP) and lectin-bound AFP (AFP-L3), have shown promise^[78]^. These markers have been studied by itself or in combination with age and gender in what is called the GALAD score. Moreover, cell free DNA (cfDNA) - DNA released by tumor cells that can be detected in peripheral blood samples; has emerged as a novel tool with high potential. Indeed, methylation of several genes (i.e., HOXA1, TSPLY5, PFKP, *etc*.) in cfDNA have been shown to have a reasonable predictive value for HCC^[79,80]^. The combination of DNA mutations and cancer-associated proteins is a more exciting recent development in detecting HCC. In one study, a panel of four genes (TP53, CTNNB1, AXIN1, TERT), AFP, and DCP were used to distinguish. HCC patients, with 85% sensitivity and 93% specificity in the training cohort^[81]^. An alternative group of biomarkers, include tumor associated antigens and antibodies such as squamous cell carcinoma antigen (SCCA) - and these biomarkers show potential, not only as a marker of early detection of HCC but may also help uncover new drug targets^[82]^.

Another promising method of early cancer detection is based on advanced imaging techniques. These include the use of artificial intelligence for digital extraction of high dimensional quantitative data from imaging^[83]^. Additional modalities to enhance imaging involve utilizing near-infrared indocyanine green fluorescence (NIRF) probes which bind to surface proteins, due to its faster uptake and deeper penetration depth^[84]^. These techniques may improve early radiologic characterization and diagnostic accuracy.

None of these biomarkers and modalities, however, have been studied in the HIV population. Moreover, the reproducibility of these tests and access to diagnostic equipment with enough capacity to measure them in resource-limited settings represent a substantial problem.

**Figure 3.**
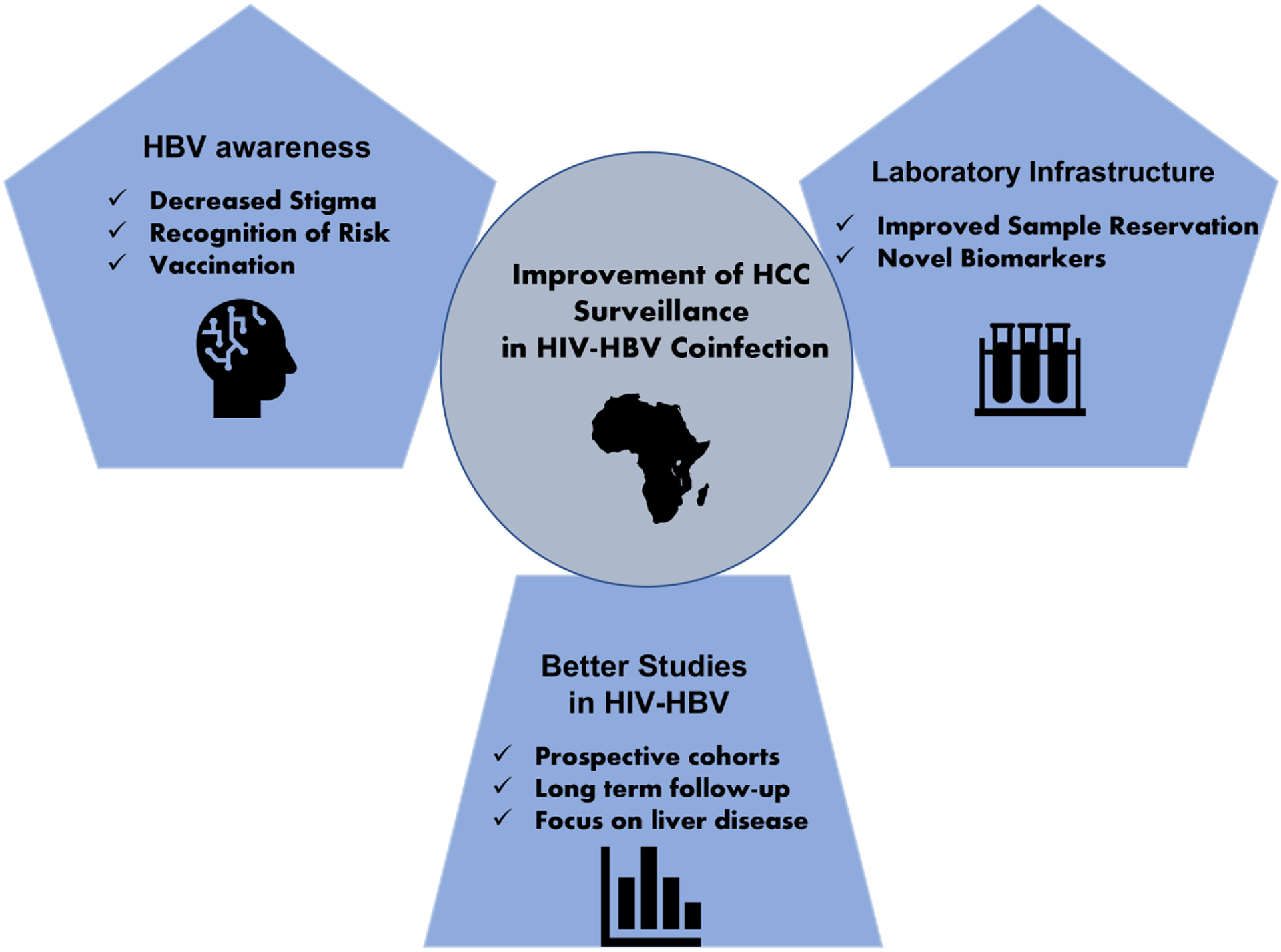
Major changes needed to improve surveillance of HCC in HIV-HBV co-infected individuals in Africa. Flow chart describing the three aspects the authors deem necessary to improve HCC surveillance in HIV-HBV coinfection, including increased awareness of HBV disease, upgrading of laboratory infrastructure, and advancement of relevant cohort studies.

Overall, three major changes are needed to improve surveillance of HCC in HIV-HBV coinfected individuals [Figure 3]. First, awareness of the risk of HCC imposed by HBV, with or without HIV should increase dramatically continent-wide. Some countries have shown an important progress in these areas with awareness stimulation in television and radio programs, as well as outreach in rural regions^[85,86]^. However, further work in this area is critical to decrease stigma, increase understanding of the risks of HBV and improve vaccination rates, with reach of vaccination to rural areas as well as implementation of HBV birth-dose. Second, more investment in laboratory infrastructure should be considered. As biomarkers in blood become better and more accurate, samples are likely to become also more complex to process and more likely to require certain stability in the temperature chain when transporting. The African continent can benefit tremendously from novel biomarkers for HCC, and other cancers, but only if the proper infrastructure is present to implement them. Third, carefully designed, multicenter, well-controlled prospective cohort studies on HIV-HBV population focused on HCC are needed in the region. This is imperative, as most of the limited information on this lethal combination to the liver, originates in North America, Europe and Asia. Studies on HBV mono-infection show that the risk and features of HCC in Africa are quite different than that of the rest of the globe^[87,88]^. We should expect that the same is true for HIV-HBV coinfection and HCC. Therefore, proper studies specifically tailored for African populations and properly powered are a must.

All these changes needed for HCC surveillance improvement in HBV-HIV are reachable, and indeed scientists, governmental and non-governmental organizations are working tirelessly to achieve them. However, these important aspects will only show dramatic improvement when applied broadly in the continent, rather than in pockets of excellence.

## References

[1] Akinyemiju T, Abera S, Ahmed M, ; Global Burden of Disease Liver Cancer Collaboration. The burden of primary liver cancer and underlying etiologies from 1990 to 2015 at the global, regional, and national level: results from the global burden of disease study 2015. JAMA Oncol 2017;3:1683–91. 28983565 10.1001/jamaoncol.2017.3055 PMC5824275

[2] Perz JF, Armstrong GL, Farrington LA, Hutin YJ, Bell BP. The contributions of hepatitis B virus and hepatitis C virus infections to cirrhosis and primary liver cancer worldwide. J Hepatol 2006;45:529–38. 16879891 10.1016/j.jhep.2006.05.013

[3] WHO. Global progress report on HIV, viral hepatitis and sexually transmitted infections, 2021. Available from: https://www.who.int/publications/i/item/9789240027077 [Last accessed on 12 Oct 2022].

[4] Joshi D, O’grady J, Dieterich D, Gazzard B, Agarwal K. Increasing burden of liver disease in patients with HIV infection. The Lancet 2011;377:1198–209. 10.1016/S0140-6736(10)62001-6 21459211

[5] Puoti M, Bruno R, Soriano V, ; HIV HCC Cooperative Italian-Spanish Group. Hepatocellular carcinoma in HIV-infected patients: epidemiological features, clinical presentation and outcome. AIDS 2004;18:2285–93. 15577541 10.1097/00002030-200411190-00009

[6] Saud LRC, Chagas AL, Maccali C, Hepatocellular carcinoma in patients coinfected with hepatitis B or C and HIV: more aggressive tumor behavior? Eur J Gastroenterol Hepatol 2021;33:583–8. 33560682 10.1097/MEG.0000000000002057 PMC9446514

[7] El-Kassas M, Elbadry M. Hepatocellular carcinoma in Africa: challenges and opportunities. Front Med (Lausanne) 2022;9:899420. 35814750 10.3389/fmed.2022.899420 PMC9263092

[8] Mbaga DS, Kenmoe S, Kengne-Ndé C, Hepatitis B, C and D virus infections and risk of hepatocellular carcinoma in Africa: a meta-analysis including sensitivity analyses for studies comparable for confounders. PLoS One 2022;17:e0262903. 35061846 10.1371/journal.pone.0262903 PMC8782350

[9] Tanon A, Jaquet A, Ekouevi DK, ; IeDEA West Africa Collaboration. The spectrum of cancers in West Africa: associations with human immunodeficiency virus. PLoS One 2012;7:e48108. 23144732 10.1371/journal.pone.0048108 PMC3483170

[10] Maponga TG, Glashoff RH, Vermeulen H, Hepatitis B virus-associated hepatocellular carcinoma in South Africa in the era of HIV. BMC Gastroenterol 2020;20:226. 32660431 10.1186/s12876-020-01372-2 PMC7359588

[11] Singal AG, Pillai A, Tiro J. Early detection, curative treatment, and survival rates for hepatocellular carcinoma surveillance in patients with cirrhosis: a meta-analysis. PLoS Med 2014;11:e1001624. 24691105 10.1371/journal.pmed.1001624 PMC3972088

[12] Rodríguez de Lope C, Reig M, Matilla A, ; en representación del Grupo de Estudio de Cáncer Hepático (GECH). Clinical characteristics of hepatocellular carcinoma in Spain. Comparison with the 2008–2009 period and analysis of the causes of diagnosis out of screening programs. Analysis of 686 cases in 73 centers. Med Clin (Barc) 2017;149:61–71. 28279536 10.1016/j.medcli.2016.12.048

[13] Alter MJ. Epidemiology of viral hepatitis and HIV co-infection. J Hepatol 2006;44:S6–9. 16352363 10.1016/j.jhep.2005.11.004

[14] Leumi S, Bigna JJ, Amougou MA, Ngouo A, Nyaga UF, Noubiap JJ. Global Burden of Hepatitis B Infection in People Living With Human Immunodeficiency Virus: A Systematic Review and Meta-analysis. Clin Infect Dis 2020;71:2799–806. 31813969 10.1093/cid/ciz1170

[15] Statista.. HIV prevalence country ranking 2018. Available from: https://www.statista.com/statistics/270209/countries-with-the-highest-global-hiv-prevalence/ [Last accessed on 12 Oct 2022].

[16] World Health Organization. Global hepatitis report, 2017. Available from: https://www.who.int/publications/i/item/9789241565455 [Last accessed on 7 Jun 2022].

[17] Deressa T, Damtie D, Fonseca K, The burden of hepatitis B virus (HBV) infection, genotypes and drug resistance mutations in human immunodeficiency virus-positive patients in Northwest Ethiopia. PLoS One 2017;12:e0190149. 29281718 10.1371/journal.pone.0190149 PMC5744989

[18] Ocama P, Seremba E, Apica B, Opio K. Hepatitis B and HIV co-infection is still treated using lamivudine-only antiretroviral therapy combination in Uganda. Afr Health Sci 2015;15:328–33. 26124776 10.4314/ahs.v15i2.4 PMC4480502

[19] Kilonzo SB, Gunda DW, Kashasha F, Mpondo BC. Liver fibrosis and hepatitis B coinfection among art naïve HIV-infected patients at a tertiary level hospital in northwestern Tanzania: a cross-sectional study. J Trop Med 2017;2017:5629130. 10.1155/2017/5629130 PMC5554579 28828009

[20] Yousif M, Mudawi H, Hussein W, Genotyping and virological characteristics of hepatitis B virus in HIV-infected individuals in Sudan. Int J Infect Dis 2014;29:125–32. 25449246 10.1016/j.ijid.2014.07.002

[21] Magoro T, Gachara G, Mavhandu L, Serologic and genotypic characterization of hepatitis B virus in HIV-1 infected patients from South West and Littoral Regions of Cameroon. Virol J 2016;13:178. 27769271 10.1186/s12985-016-0636-x PMC5073451

[22] Stabinski L, OʼConnor S, Barnhart M, Kahn RJ, Hamm TE. Prevalence of HIV and hepatitis B virus co-infection in sub-Saharan Africa and the potential impact and program feasibility of hepatitis B surface antigen screening in resource-limited settings. J Acquir Immune Defic Syndr 2015;68 Suppl 3:S274–85. 25768867 10.1097/QAI.0000000000000496 PMC10426262

[23] Kenfack-Momo R, Kenmoe S, Takuissu GR, Epidemiology of hepatitis B virus and/or hepatitis C virus infections among people living with human immunodeficiency virus in Africa: a systematic review and meta-analysis. PLoS One 2022;17:e0269250. 35639675 10.1371/journal.pone.0269250 PMC9154112

[24] Spearman CW, Afihene M, Ally R, Hepatitis B in sub-Saharan Africa: strategies to achieve the 2030 elimination targets. The Lancet Gastroenterology & Hepatology 2017;2:900–9. 29132759 10.1016/S2468-1253(17)30295-9

[25] Weber R, Sabin CA, Friis-Møller N, Liver-related deaths in persons infected with the human immunodeficiency virus: the D:A:D study. Arch Intern Med 2006;166:1632–41. 16908797 10.1001/archinte.166.15.1632

[26] Yang JD, Hainaut P, Gores GJ, Amadou A, Plymoth A, Roberts LR. A global view of hepatocellular carcinoma: trends, risk, prevention and management. Nat Rev Gastroenterol Hepatol 2019;16:589–604. 31439937 10.1038/s41575-019-0186-y PMC6813818

[27] Tuyama AC, Hong F, Saiman Y, Human immunodeficiency virus (HIV)-1 infects human hepatic stellate cells and promotes collagen I and monocyte chemoattractant protein-1 expression: implications for the pathogenesis of HIV/hepatitis C virus-induced liver fibrosis. Hepatology 2010;52:612–22. 20683959 10.1002/hep.23679 PMC2917256

[28] Debes JD, Bohjanen PR, Boonstra A. Mechanisms of accelerated liver fibrosis progression during HIV infection. J Clin Transl Hepatol 2016;4:328–35. 28097102 10.14218/JCTH.2016.00034 PMC5225153

[29] Thornton AC, Jose S, Bhagani S, ; UK Collaborative HIV cohort (UK CHIC) steering committee. Hepatitis B, hepatitis C, and mortality among HIV-positive individuals. AIDS 2017;31:2525–32. 28926400 10.1097/QAD.0000000000001646 PMC5690308

[30] Thio CL, Seaberg EC, Skolasky R, HIV-1, hepatitis B virus, and risk of liver-related mortality in the multicenter cohort study (MACS). The Lancet 2002;360:1921–6. 10.1016/s0140-6736(02)11913-1 12493258

[31] Colin JF, Cazals-Hatem D, Loriot MA, Influence of human immunodeficiency virus infection on chronic hepatitis B in homosexual men. Hepatology 1999;29:1306–10. 10094979 10.1002/hep.510290447

[32] Pinato DJ, Dalla Pria A, Sharma R, Bower M. Hepatocellular carcinoma: an evolving challenge in viral hepatitis and HIV coinfection. AIDS 2017;31:603–11. 28121711 10.1097/QAD.0000000000001422

[33] Singh KP, Crane M, Audsley J, Avihingsanon A, Sasadeusz J, Lewin SR. HIV-hepatitis B virus coinfection: epidemiology, pathogenesis, and treatment. AIDS 2017;31:2035–52. 28692539 10.1097/QAD.0000000000001574 PMC5661989

[34] Kabarambi A, Balinda S, Abaasa A, Cogill D, Orrell C. Determinants and reasons for switching anti-retroviral regimen among HIV-infected youth in a large township of South Africa (2002–2019). AIDS Res Ther 2022;19:32. 35765006 10.1186/s12981-022-00453-4 PMC9237968

[35] Hernández-ramírez RU, Shiels MS, Dubrow R, Engels EA. Cancer risk in HIV-infected people in the USA from 1996 to 2012: a population-based, registry-linkage study. The Lancet HIV 2017;4:e495–504. 28803888 10.1016/S2352-3018(17)30125-X PMC5669995

[36] 1993 revised classification system for HIV infection and expanded surveillance case definition for AIDS among adolescents and adults. MMWR Recomm Rep 1992;41(RR-17):1–19. 1361652

[37] Epeldegui M, Vendrame E, Martínez-Maza O. HIV-associated immune dysfunction and viral infection: role in the pathogenesis of AIDS-related lymphoma. Immunol Res 2010;48:72–83. 20717742 10.1007/s12026-010-8168-8 PMC3640300

[38] Martínez-Maza O, Breen EC. B-cell activation and lymphoma in patients with HIV. Curr Opin Oncol 2002;14:528–32. 12192272 10.1097/00001622-200209000-00009

[39] Althoff KN, Stewart CN, Humes E, The shifting age distribution of people with HIV using antiretroviral therapy in the United States. AIDS 2022;36:459–71. 34750289 10.1097/QAD.0000000000003128 PMC8795488

[40] Anugwom CM, Allaire M, Akbar SMF, Hepatitis B-related hepatocellular carcinoma: surveillance strategy directed by immune-epidemiology. Hepatoma Res 2021;7:23. 33884303 10.20517/2394-5079.2021.06 PMC8057710

[41] Venkataramani M, Hutton N, Colombani P, Anders RA, Agwu AL. Hepatocellular carcinoma in a teenager with perinatally acquired HIV Infection without hepatitis B or C coinfection: a case report. AIDS Patient Care STDS 2010;24:693–6. 20969466 10.1089/apc.2010.0038 PMC2994537

[42] Giordano TP, Kramer JR, Souchek J, Richardson P, El-Serag HB. Cirrhosis and hepatocellular carcinoma in HIV-infected veterans with and without the hepatitis C virus: a cohort study, 1992–2001. Arch Intern Med 2004;164:2349–54. 15557414 10.1001/archinte.164.21.2349

[43] Jaquet A, Odutola M, Ekouevi DK, ; IeDEA West Africa Collaboration. Cancer and HIV infection in referral hospitals from four West African countries. Cancer Epidemiol 2015;39:1060–5. 26375806 10.1016/j.canep.2015.09.002 PMC4679441

[44] Kramer JR, Kowalkowski MA, Duan Z, Chiao EY. The effect of HIV viral control on the incidence of hepatocellular carcinoma in veterans with hepatitis C and HIV coinfection. J Acquir Immune Defic Syndr 2015;68:456–62. 25559606 10.1097/QAI.0000000000000494 PMC4334674

[45] Ochoa-Callejero L, Pérez-Martínez L, Rubio-Mediavilla S, Oteo JA, Martínez A, Blanco JR. Maraviroc, a CCR5 antagonist, prevents development of hepatocellular carcinoma in a mouse model. PLoS One 2013;8:e53992. 23326556 10.1371/journal.pone.0053992 PMC3541191

[46] García-Samaniego J Hepatocellular carcinoma in HIV-infected patients with chronic hepatitis C. Am J Gastroenterol 2001;96:179–83. 11197250 10.1111/j.1572-0241.2001.03374.x

[47] Berretta M, Garlassi E, Cacopardo B, Hepatocellular carcinoma in HIV-infected patients: check early, treat hard. Oncologist 2011;16:1258–69. 21868692 10.1634/theoncologist.2010-0400 PMC3228175

[48] Ryom L, Lundgren JD, De Wit S, ; D:A:D Study Group. Use of antiretroviral therapy and risk of end-stage liver disease and hepatocellular carcinoma in HIV-positive persons. AIDS 2016;30:1731–43. 26752282 10.1097/QAD.0000000000001018

[49] Yang R, Gui X, Ke H, Xiong Y, Gao S. Combination antiretroviral therapy is associated with reduction in liver fibrosis scores in patients with HIV and HBV co-infection. AIDS Res Ther 2021;18:98. 34924016 10.1186/s12981-021-00419-y PMC8684625

[50] Boyd A, Bottero J, Miailhes P, Liver fibrosis regression and progression during controlled hepatitis B virus infection among HIV-HBV patients treated with tenofovir disoproxil fumarate in France: a prospective cohort study. J Int AIDS Soc 2017;20:21426. 28362068 10.7448/IAS.20.1.21426 PMC5467614

[51] Li KW, Kramvis A, Liang S, Higher prevalence of cancer related mutations 1762T/1764A and PreS deletions in hepatitis B virus (HBV) isolated from HBV/HIV co-infected compared to HBV-mono-infected Chinese adults. Virus Res 2017;227:88–95. 27720823 10.1016/j.virusres.2016.10.002

[52] Audsley J, Littlejohn M, Yuen L, HBV mutations in untreated HIV-HBV co-infection using genomic length sequencing. Virology 2010;405:539–47. 20655563 10.1016/j.virol.2010.06.038 PMC2935930

[53] Stella L, Santopaolo F, Gasbarrini A, Pompili M, Ponziani FR. Viral hepatitis and hepatocellular carcinoma: From molecular pathways to the role of clinical surveillance and antiviral treatment. World J Gastroenterol 2022;28:2251–81. 35800182 10.3748/wjg.v28.i21.2251 PMC9185215

[54] Chidambaranathan-reghupaty S, Fisher PB, Sarkar D. Hepatocellular carcinoma (HCC): epidemiology, etiology and molecular classification. Adv Cancer Res 2021;149:1–61. 33579421 10.1016/bs.acr.2020.10.001 PMC8796122

[55] Rossotti R, Merli M, Mazzarelli C, Similar survival but higher and delayed hepatocellular carcinoma recurrence in HIV-positive compared to negative cirrhotics undergoing liver transplantation. Dig Liver Dis; 2022:S1590-8658(22)00500. 10.1016/j.dld.2022.05.001 35644890

[56] Cunha L, Carrilho C, Bhatt N, Hepatocellular carcinoma: Clinical-pathological features and HIV infection in Mozambican patients. Cancer Treat Res Commun 2019;19:100129. 30903933 10.1016/j.ctarc.2019.100129 PMC6504939

[57] Okeke E, Mark Davwar P, Mullen B, The impact of HIV on hepatocellular cancer survival in Nigeria. Trop Med Int Health 2021;26:335–42. 33244817 10.1111/tmi.13532

[58] Tassachew Y, Abebe T, Belyhun Y, Prevalence of HIV and Its Co-infection with hepatitis B/C virus among chronic liver disease patients in Ethiopia. Hepat Med 2022;14:67–77. 35591850 10.2147/HMER.S365443 PMC9113656

[59] Jaquet A, Tchounga B, Tanon A, Etiology of hepatocellular carcinoma in West Africa, a case-control study. Int J Cancer 2018;143:869–77. 29569722 10.1002/ijc.31393 PMC6041181

[60] Shiels MS, Engels EA. Evolving epidemiology of HIV-associated malignancies. Curr Opin HIV AIDS 2017;12:6–11. 27749369 10.1097/COH.0000000000000327 PMC5240042

[61] Marrero JA, Kulik LM, Sirlin CB, Diagnosis, staging, and management of hepatocellular carcinoma: 2018 practice guidance by the American association for the study of liver diseases. Hepatology 2018;68:723–50. 29624699 10.1002/hep.29913

[62] Méndez-Sánchez N, Ridruejo E, Alves de Mattos A, Latin American association for the study of the liver (LAASL) clinical practice guidelines: management of hepatocellular carcinoma. Ann Hepatol 2014;13(Suppl 1):S4–40. 24998696

[63] Association for the Study of the Liver. Electronic address: easloffice@easloffice.eu. Corrigendum to EASL clinical practice guidelines: management of hepatocellular carcinoma. J Hepatol 2019;70:817. 30739718 10.1016/j.jhep.2019.01.020

[64] Beauchamp E, Rollet K, Walmsley S, Wong DK, Cooper C, Klein MB; Canadian Co-infection Cohort Study (CTN222). Missed opportunities for hepatocellular carcinoma screening in an HIV/hepatitis C virus-coinfected cohort. Clin Infect Dis 2013;57:1339–42. 23899675 10.1093/cid/cit491

[65] Lim C, Goutte N, Gervais A, Standardized care management ensures similar survival rates in HIV-positive and HIV-negative patients with hepatocellular carcinoma. J Acquir Immune Defic Syndr 2012;61:581–7. 22918160 10.1097/QAI.0b013e31826ebdc7

[66] Patel N, Post FA. Surveillance for hepatocellular carcinoma in people of African ancestry with HIV and Hepatitis B. Int J STD AIDS 2022;33:202–4. 34546112 10.1177/09564624211042828

[67] Willemse S, Smit C, Sogni P, ; Hepatocellular Carcinoma Screening Project Working Group for the Collaboration of Observational HIV on behalf of Epidemiological Research Europe (COHERE) In EuroCoord. Low compliance with hepatocellular carcinoma screening guidelines in hepatitis B/C virus co-infected HIV patients with cirrhosis. J Viral Hepat 2019;26:1224–8. 31136059 10.1111/jvh.13146 PMC6851829

[68] Hearn B, Chasan R, Bichoupan K, Low adherence of HIV providers to practice guidelines for hepatocellular carcinoma screening in HIV/hepatitis B coinfection. Clin Infect Dis 2015;61:1742–8. 26240206 10.1093/cid/civ654 PMC4809983

[69] Yang JD, Mohamed EA, Aziz AOA, Characteristics, management, and outcomes of patients with hepatocellular carcinoma in Africa: a multicountry observational study from the Africa Liver Cancer Consortium. The Lancet Gastroenterology & Hepatology 2017;2:103–11. 28403980 10.1016/S2468-1253(16)30161-3

[70] Kedar Mukthinuthalapati VVP, Sewram V, Ndlovu N, Hepatocellular Carcinoma in Sub-Saharan Africa. JCO Glob Oncol 2021;7:756–66. 34043413 10.1200/GO.20.00425 PMC8457845

[71] Kew MC. Hepatic iron overload and hepatocellular carcinoma. Liver Cancer 2014;3:31–40. 24804175 10.1159/000343856 PMC3995380

[72] Okeke E, Davwar PM, Roberts L, Epidemiology of liver cancer in Africa: current and future trends. Semin Liver Dis 2020;40:111–23. 31726474 10.1055/s-0039-3399566

[73] Ocama P, Opio KC, Kagimu M, Seremba E, Wabinga H, Colebunders R. Hepatitis B virus and HIV infection among patients with primary hepatocellular carcinoma in Kampala, Uganda. Afr Health Sci 2011;11 Suppl 1:S20–3. 22135639 10.4314/ahs.v11i3.70065 PMC3220119

[74] Gomaa A, Rewisha E, Saleh S, Waked I. Changing presenting features of hepatocellular carcinoma in Egyptian patients. 7th International Liver Cancer Association (ILCA) Annual Conference 2013, September 13–15; Washington, DC, USA: Abstract P-077. DOI

[75] Riebensahm C, Chitundu H, Muula G, ; IeDEA-Southern Africa. Screening for hepatocellular carcinoma among adults with HIV/HBV co-infection in Zambia: a pilot study. Int J Infect Dis 2022;116:391–6. 34952210 10.1016/j.ijid.2021.12.338 PMC9912380

[76] Tzartzeva K, Obi J, Rich NE, Surveillance imaging and alpha fetoprotein for early detection of hepatocellular carcinoma in patients with cirrhosis: a meta-analysis. Gastroenterology 2018;154:1706–1718.e1. 29425931 10.1053/j.gastro.2018.01.064 PMC5927818

[77] Stewart KA, Navarro SM, Kambala S, Trends in ultrasound use in low and middle income countries: a systematic review. Int J MCH AIDS 2020;9:103–20. 32123634 10.21106/ijma.294 PMC7031872

[78] Berhane S, Toyoda H, Tada T, Role of the GALAD and BALAD-2 serologic models in diagnosis of hepatocellular carcinoma and prediction of survival in patients. Clin Gastroenterol Hepatol 2016;14:875–886.e6. 26775025 10.1016/j.cgh.2015.12.042

[79] Kisiel JB, Dukek BA, V S R Kanipakam R, Hepatocellular carcinoma detection by plasma methylated dna: discovery, phase I pilot, and phase II clinical validation. Hepatology 2019;69:1180–92. 30168613 10.1002/hep.30244 PMC6429916

[80] Chalasani NP, Ramasubramanian TS, Bhattacharya A, A novel blood-based panel of methylated DNA and protein markers for detection of early-stage hepatocellular carcinoma. Clin Gastroenterol Hepatol 2021;19:2597–2605.e4. 32889146 10.1016/j.cgh.2020.08.065

[81] Qu C, Wang Y, Wang P, Detection of early-stage hepatocellular carcinoma in asymptomatic HBsAg-seropositive individuals by liquid biopsy. Proc Natl Acad Sci U S A 2019;116:6308–12. 30858324 10.1073/pnas.1819799116 PMC6442629

[82] Pontisso P, Calabrese F, Benvegnù L, Overexpression of squamous cell carcinoma antigen variants in hepatocellular carcinoma. Br J Cancer 2004;90:833–7. 14970861 10.1038/sj.bjc.6601543 PMC2410161

[83] Mokrane FZ, Lu L, Vavasseur A, Radiomics machine-learning signature for diagnosis of hepatocellular carcinoma in cirrhotic patients with indeterminate liver nodules. Eur Radiol 2020;30:558–70. 31444598 10.1007/s00330-019-06347-w

[84] Zhang W, Hu Z, Tian J, Fang C. A narrative review of near-infrared fluorescence imaging in hepatectomy for hepatocellular carcinoma. Ann Transl Med 2021;9:171. 33569473 10.21037/atm-20-5341 PMC7867918

[85] Quadri NS, Shah SM, Rodin H, Debes JD. Promoting hepatitis b awareness: evaluating an educational approach through health care workers in Tanzania. Ann Glob Health 2021;87:22. 33665144 10.5334/aogh.3045 PMC7908926

[86] Quadri NS, Debes JD. The Waiting Room Project: An Approach to Community Health Education in Hepatitis B. Am J Trop Med Hyg 2020;103:537. 32653047 10.4269/ajtmh.20-0232 PMC7356481

[87] Yang JD, Gyedu A, Afihene MY, ; Africa Network for Gastrointestinal and Liver Diseases. Hepatocellular carcinoma occurs at an earlier age in Africans, particularly in association with chronic hepatitis B. Am J Gastroenterol 2015;110:1629–31. 26618430 10.1038/ajg.2015.289

[88] Sultan A, Anugwom CM, Wondifraw Z, Braimoh GA, Bane A, Debes JD. Single center analysis of therapy and outcomes of hepatocellular carcinoma in Sub-Saharan Africa. Expert Rev Gastroenterol Hepatol 2020;14:1007–11. 32730120 10.1080/17474124.2020.1802246 PMC7544626

